# Point-of-Care Disease Screening in Primary Care Using Saliva: A Biospectroscopy Approach for Lung Cancer and Prostate Cancer

**DOI:** 10.3390/jpm13111533

**Published:** 2023-10-26

**Authors:** Francis L. Martin, Camilo L. M. Morais, Andrew W. Dickinson, Tarek Saba, Thomas Bongers, Maneesh N. Singh, Danielle Bury

**Affiliations:** 1 Biocel UK Ltd., Hull HU10 6TS, UK; mnsingh@biocel.uk; 2 Department of Cellular Pathology, Blackpool Teaching Hospitals NHS Foundation Trust, Whinney Heys Road, Blackpool FY3 8NR, UK; aw.dickinson@hotmail.com (A.W.D.); dr.saba@nhs.net (T.S.); thomas.bongers@nhs.net (T.B.); 3 Center for Education, Science and Technology of the Inhamuns Region, State University of Ceará, Tauá 63660-000, Brazil; camilomorais1@gmail.com; 4 Chesterfield Royal Hospital, Chesterfield Road, Calow, Chesterfield S44 5BL, UK

**Keywords:** ATR-FTIR spectroscopy, chemometrics, “dip” test, cancer screening, saliva, swab

## Abstract

Saliva is a largely unexplored liquid biopsy that can be readily obtained noninvasively. Not dissimilar to blood plasma or serum, it contains a vast array of bioconstituents that may be associated with the absence or presence of a disease condition. Given its ease of access, the use of saliva is potentially ideal in a point-of-care screening or diagnostic test. Herein, we developed a swab “dip” test in saliva obtained from consenting patients participating in a lung cancer-screening programme being undertaken in north-west England. A total of 998 saliva samples (31 designated as lung-cancer positive and 17 as prostate-cancer positive) were taken in the order in which they entered the clinic (i.e., there was no selection of participants) during the course of this prospective screening programme. Samples (sterile Copan blue rayon swabs dipped in saliva) were analysed using attenuated total reflection Fourier-transform infrared (ATR-FTIR) spectroscopy. In addition to unsupervised classification on resultant infrared (IR) spectra using principal component analysis (PCA), a range of feature selection/extraction algorithms were tested. Following preprocessing, the data were split between training (70% of samples, 22 lung-cancer positive versus 664 other) and test (30% of samples, 9 lung-cancer positive versus 284 other) sets. The training set was used for model construction and the test set was used for validation. The best model was the PCA-quadratic discriminant analysis (QDA) algorithm. This PCA-QDA model was built using 8 PCs (90.4% of explained variance) and resulted in 93% accuracy for training and 91% for testing, with clinical sensitivity at 100% and specificity at 91%. Additionally, for prostate cancer patients amongst the male cohort (n = 585), following preprocessing, the data were split between training (70% of samples, 12 prostate-cancer positive versus 399 other) and test (30% of samples, 5 prostate-cancer positive versus 171 other) sets. A PCA-QDA model, again the best model, was built using 5 PCs (84.2% of explained variance) and resulted in 97% accuracy for training and 93% for testing, with clinical sensitivity at 100% and specificity at 92%. These results point to a powerful new approach towards the capability to screen large cohorts of individuals in primary care settings for underlying malignant disease.

## 1. Introduction

There is strong evidence that screening for preinvasive or early disease results in earlier detection of cancer and dramatically improves outcomes [1]. This has been singularly noted with the smear test for cervical cancer, mammography for breast cancer and the faecal immunochemical test for bowel cancer. There is increasing interest in the use of liquid biopsies in analytical tests that may evidence the presence of malignancy at a known, or even unknown, target site [2]. Such tests, if applicable, in a low-cost and high-throughput fashion with a low false-positive rate could significantly reduce the burden of advanced disease diagnosis. It would be a transformative step in cancer management.

Saliva is a readily accessible liquid biopsy and can be sourced either by a pharyngeal swab [3] or by getting the patient to spit into a sterile collection vessel [4]. Daily, humans produce up to 1.5 litres of saliva, a biologically rich biofluid [5]. Although blood plasma or serum has been traditionally the liquid biopsy of choice for clinical biochemistry testing, obtaining such samples is not noninvasive and, in some instances, especially with older or chronically unwell individuals, difficult or painful to obtain. The saliva proteome markedly overlaps with that of the plasma proteome [6,7,8]. For instance, in pain management, salivary biomarkers such as cortisol, α-amylase or immunoglobulins are detectable and appear to fluctuate depending on the state of the patient [9]. Given its ready accessibility, a tool or approach that could readily analyse saliva for the presence or absence of high-burden malignant diseases would have enormous potential in a point-of-care clinical setting.

Within a prospective study of patients attending the Blackpool Targeted Lung Health Check [10], we have collected saliva samples from the first 1000 patients recruited to this trial. Recruits were preselected through local primary care records based on multiple factors, including age and smoking history, primarily because they were deemed ‘at risk’ of lung cancer. Following initial health checks (which included getting demographic data and information on other morbidities such as breast cancer in females and prostate cancer in males), patients that trigger a need to perform a low-radiation dose computed tomography (CT) scan for further investigations were consented to take part in this study. The research nurse undertaking this initial assessment consented to recruits for involvement in the screening pilot. These patients were then requested to provide saliva for testing by spitting into a sterile universal container, with the primary aim of examining the use of such saliva analysis as a rapid screening tool for the presence of underlying lung cancer. The possibility of using saliva to screen for other morbidities was also examined, if numbers permitted. The saliva was tested on a portable IR spectrometer [4]. Chemometric analysis to develop predictive models to allow the determination of sensitivities and specificities for saliva for the diagnosis of lung cancer or prostate cancer (in recruited males) was undertaken. Lung cancer and prostate cancer are common morbidities that would be expected to occur at a reasonable prevalence in an aged population such as this.

The application of reagent-free and nondestructive spectrochemical methods, such as attenuated total reflection Fourier-transform infrared (ATR-FTIR) spectroscopy, combined with chemometrics, is gaining increasing recognition as potential screening and/or diagnostic tools in clinical settings [11]. A deviation in the fingerprint absorbance spectrum of a target biological material may be predictive of an adverse outcome, such as disease. The approach is predicated on the construction of a computational algorithm that allows one to determine with high (>75%) clinical sensitivity and specificity the possibility of an adverse diagnosis [12]. The ready applicability of liquid biopsies such as blood plasma/serum, saliva or urine in such an analytical context is obvious, and ATR-FTIR spectroscopy approaches have been used for diagnosing, screening or monitoring the progression/regression of a variety of disease conditions [13,14]. One could readily argue that saliva is the most readily accessible of the aforementioned liquid biopsies. It has previously been demonstrated possible to employ saliva analysed using ATR-FTIR spectroscopy to distinguish from normal through Barrett’s oesophagus, dysplasia up to adenocarcinoma. Within the normal versus adenocarcinoma groups, this is achieved with sensitivities of 89–100% and specificities of 60–100% [15].

This study set out to examine whether a “dip” test based on the spectrochemical analysis of saliva with subsequent chemometrics on derived infrared (IR) spectra could be used to generate a noninvasive screening approach for lung cancer. This study was nested within the National Lung Cancer Screening Pilot within the north-west region of England. This region is known to have a large-scale prevalence of common cancers, including those of the lung, breast and prostate, probably due to the poor lifestyle associated with high levels of social deprivation in coastal towns. The CancerResearch UK website (www.cancerresearchuk.org/, accessed 16 October 2023), in the 2016–2018 time frame in the UK, reports that there were 48,549 cases and 34,771 deaths from lung cancer, whereas there were 52,254 cases and 12,039 deaths from prostate cancer. Based on our initial pilot [4], we set out to further explore whether a swab-based “dip” test whereby a plain sterile rayon-tipped swab dipped in a saliva sample provided by a patient and then spectrochemically analysed using a FTIR Spectrometer equipped with an ATR ZnSe crystal would generate IR spectra containing sufficient features so that a computational algorithm could be developed to screen for disease. Our initial objective was to determine if the method was robust enough to screen for underlying lung cancer. However, given the age range of our study participants and the fact that we had demographic information on the presence or absence of other diseases such as prostate cancer (in men) or breast cancer (in women), we also explored the possibility of screening for these other conditions. There were insufficient numbers of females and cases to robustly explore a screening outcome for breast cancer. However, there were a sufficient number of males recruited into the study and prostate cancer cases. A number of feature extraction chemometric approaches were explored to determine if an algorithm approach built and validated on known outcomes could deliver a screening of unknowns (i.e., IR spectra of blinded saliva samples) with high clinical sensitivity and specificity. If successful, such an approach would carry enormous potential as an inexpensive, easy-to-use and rapid point-of-care screening tool for chronic conditions. Our aim is to validate this biospectroscopy approach in clinical settings.

## 2. Materials and Methods

### 2.1. Lung Cancer Screening Programme and Participant Recruitment

This study was carried out in agreement with the Helsinki Declaration and full ethical approval was obtained (HRA IRAS ref: 276081; REC ref: 20/PR/0390; London Bridge REC). All procedures and possible risks were explained to participants before they provided written consent. The study was nested in a prospective study of people invited to attend the National Lung Cancer Screening Pilot in the Blackpool area of north-west England. These potential participants were preselected based on multiple factors, including age and smoking history, to be deemed ‘at risk’ of lung cancer or prostate cancer. Once they had undergone health checks, those participants who triggered a CT scan for further investigation consented, if willing, to take part in this study. This was performed by the nurse undertaking the initial assessment and consent for involvement in the screening pilot. The rationale for this approach was to provide a mixture of both suspected cancer and noncancer patients. All participants had a CT scan, and those that exhibited no lung or prostate lesions were immediately assigned to the benign group. A visible lesion triggered an urgent oncology referral. Participants who underwent surgery were proven to have cancer following histopathology undertaken by a consultant histopathologist. A small number of participants had radiotherapy; these were also assigned as cancer. Additionally, some participants sent for oncology referral had benign lesions; these individuals were assigned to the benign group. All participants were followed for up to 2 years in order to validate these outcomes. A total of 998 saliva samples (from which, 31 designated as lung-cancer positive and 17 as prostate-cancer positive) were randomly taken in the order in which they entered the clinic (i.e., there was no selection of participants in order to avoid bias) during the course of this prospective lung and prostate cancer-screening programme.

### 2.2. Saliva Collection and Swab Analysis

For all participants, demographic data (age, gender, pre-existing medical conditions, symptoms, date of symptoms’ onset—see Supplementary Materials) were collected for NHS records; these will be accessible as the study progresses and more outcomes are known. Once consent has been given, participants were requested to provide saliva for testing by spitting into a sterile universal container. Samples were transported to the laboratory within 24 h where they were frozen at −20 °C until preparation for analysis. Towards spectral analysis, a plain sterile rayon-tipped swab (Ref no.: 155C; Copan, Italy) was placed in the thawed (at room temperature) saliva sample to be tested and mixed prior to spectral interrogation of the swab. The swab was applied directly to the ATR ZnSe crystal for spectral analysis—this was found to be an extremely convenient means of handling this biological material. Whilst there are contributing peaks from the swab, our objective was solely to develop a technique capable of giving a yes/no answer to the possibility or not of lung or prostate cancer being present.

### 2.3. ATR-FTIR Spectral Analyses of Swabs

FTIR spectra data (wavenumber range 4000–650 cm^−1^) for each swab was obtained by directly placing the saliva swab on a portable Agilent Cary 630 FTIR Spectrometer equipped with an ATR ZnSe crystal (Agilent, Santa Clara, CA, USA) and Microlab PC software run from a dedicated computer laptop. Each whole spectrum contains 1798 points (1.86 cm^−1^ spectral resolution). For every ATR-FTIR spectroscopic measurement, three spectra were obtained from each saliva swab. Each swab analysis was performed with 32 coadditions, interspersed with 32 background scans. After each analysis, the swab was removed from the crystal and the crystal was cleaned with miliQ water (Merck, Rahway, NJ, USA) and 70% alcohol, thus avoiding intersample contamination.

### 2.4. Computational Analysis: Preprocessing and Chemometrics

All data analytics were performed using MATALB R0214b (MathWorks, Inc., Natick, MA, USA) with the aid of the PLS Toolbox version 7.9.3 (Eigenvector Research, Inc., Manson, WA, USA) and lab-made routines. Spectral preprocessing for data analysis consisted of the Savitzky-Golay (SG) 2nd derivative (window of 9 points, 2nd-order polynomial fitting) followed by vector normalisation. The SG 2nd derivative performs a combined smoothing and derivative operation in the data, where the smoothing corrects for random noise and the 2nd derivative corrects for baseline distortions. Vector normalisation was applied to correct for physical differences between samples such as thickness, light scattering and concentrations. Mean-centring was applied before multivariate analysis.

Principal component analysis (PCA) was used for exploratory analysis. PCA reduces the preprocessed spectral dataset into a small number of principal components (PCs), responsible for the majority of data variance. Each PC is composed of scores and loadings; the former is used to access similarity/dissimilarity patterns among samples and the latter to identify spectral features (wavenumbers), associated with class separation and therefore possible spectral biomarkers. This technique looks for inherent similarities/differences and provides a score matrix representing the overall “identity” of each sample; a loadings matrix representing the spectral profile in each PC; and a residual matrix containing the unexplained data. Score information can be used for exploratory analysis, providing possible classifications between data classes.

PCA was the method of choice for analysing swab spiked samples. It is simple, fast, and combines exploratory analysis, data reduction and feature extraction into one single method. PCA scores were used to explore overall dataset variance and any clustering, while the loadings on the selected PCs were used to derive specific biomarkers indicative of the lesion category.

In addition to PCA, another two feature selection/extraction algorithms were tested: (1) genetic algorithm (GA) [16], which is an iterative combinational algorithm inspired by Mendelian genetics wherein a set of initial variables (i.e., wavenumbers) undergo selection, cross-over combinations and mutations until the fittest selected variables, in terms of best classification, are found [17]; and, (2) partial least squares discriminant analysis (PLS-DA) [18], which is both a feature extraction and classification technique whereby a partial least squares model is applied to the preprocessed spectral data reducing the original dataset to a few number of latent variables (LVs), constructed by maximizing the covariance between the spectral data and class information, and then, a linear discriminant classifier is used to classify the groups [17].

Besides PLS-DA, classification was also performed in the PCA scores and in the GA selected variables by linear discriminant analysis (LDA), quadratic discriminant analysis (QDA) and support vector machines (SVM). Furthermore, k-nearest neighbours (KNN) was also tested to classify the preprocessed spectral data.

Both LDA and QDA are classifiers that assign samples to predefined classes based on their Mahalanobis distance to the class centre [19]. The main difference between these two methods is that LDA calculates the distance between the samples based on a pooled covariance matrix, thus assuming each class has similar variance structures; in contrast, QDA calculates the distance between the samples using the variance-covariance matrix for each class individually, thus not assuming they have similar variances [20].

SVM and KNN are supervised machine learning algorithms that classify the data in a nonlinear fashion. SVM is a binary linear classifier with a nonlinear step called kernel transformation [21]. For this, the input data is nonlinearly transformed into a feature space that maximises the distance between the classes, and then a linear classifier is applied to separate the groups [17]. KNN is a local nonparametric classifier where the samples are classified based on the “majority vote” approach, wherein a given test sample spectrum is projected onto a feature space based on the calculation of a distance or dissimilarity metric (i.e., Euclidian distance herein), and then, depending on the number of nearest surrounding neighbour training samples to this test sample, the sample is classified towards the majority observed class [17].

SVM and KNN are excellent classification methods, especially for nonlinear data; however, they are highly susceptible to under- or over-fitting if the kernel parameters are not judiciously selected, if the number of samples is small, and if they do not cover the entire feature space. In this study, several SVM kernels were tested, including the linear kernel, 2nd order polynomial kernel and the radial basis function (RBF) kernel, and both SVM kernel parameters and the *k*-value for KNN were optimised with 10-fold cross-validation. Likewise, GA was optimised using 100 generations with 200 chromosomes each. Crossover and mutation probabilities were set to 60% and 1%, respectively.

### 2.5. Model Validation

Before model construction, outliers were identified and removed from the dataset using the Hotelling T^2^ vs. Q residuals test [17]. Thereafter, the entire dataset was split between training (70%) and test (30%) sets using the Kennard-Stone (KS) algorithm [22]. The training set was used for model construction and optimisation and the test set for the final model validation, since these were samples external to the model (blind samples). Metrics, such as accuracy (AC), sensitivity (SENS), specificity (SPEC), F-score and G-score were calculated for model validation as follows:

$$AC=\frac{TP+TN}{TP+FP+T\;N+FN}$$

$$SENS=\frac{TP}{TP+FN}$$

$$SPEC=\frac{TN}{TN+FP}$$

$$F-\mathrm{score}=\frac{2\times SENS\times SPEC}{SENS+SPEC}$$

$$G-\mathrm{score}=\sqrt{SENS\times SPEC}$$

where *TP* stands for true positives; *TN* for true negatives; *FP* for false positives; and *FN* for false negatives.

## 3. Results

Saliva samples were obtained from consented participants in a lung and prostate cancer-screening programme. Following transport to the laboratory, a sterile Copan blue rayon swab was dipped in the saliva sample, whereupon the swab was then analysed on the IR spectrometer. From each saliva sample, three independent spectral measurements were taken and then averaged.

### 3.1. Lung Cancer

**Figure 1:**
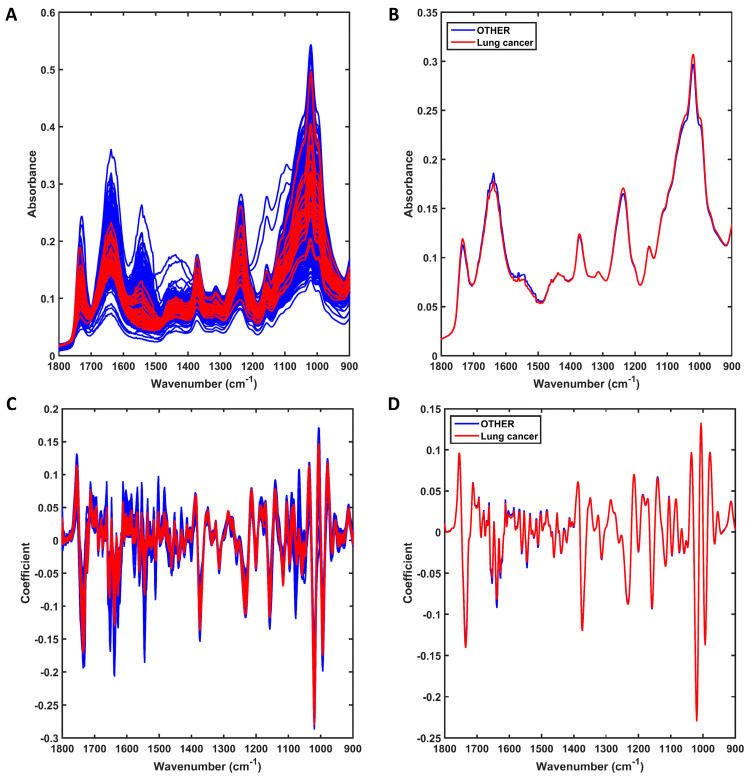
Mid-infrared (IR) spectra derived using ATR-FTIR spectroscopy. (**A**) Raw IR spectra for all sample in each group (lung cancer or other conditions [OTHER]); (**B**) average raw IR spectra for each group (lung cancer or other conditions [OTHER]). (**C**) Preprocessed (SG 2nd derivative followed by vector normalisation) IR spectra for all samples in each group (lung cancer or other conditions [OTHER]); (**D**) average preprocessed IR spectra for each group (lung cancer or other conditions [OTHER]).

The raw spectra at the fingerprint region (1800–900 cm^−1^) for the 31 lung cancer samples against all remaining patients (n = 967, OTHER) are shown in Figure 1A. Both groups of samples share similar spectral profiles, with only small differences at approximately 1500–1650 cm^−1^ and 1000–1100 cm^−1^ as shown in the average profile per class (Figure 1B). The preprocessed spectra after outlier removal are shown in Figure 1C. For this, the spectral data were preprocessed by SG 2nd derivative (window of 9 points, 2nd-order polynomial fitting), followed by vector normalisation (Figure 1C). Outliers were removed by the Hotelling T^2^ vs. Q residuals test, where 19 outliers were identified in the other-conditions group (see Supplementary Figure S1). No LG outlier was observed. Therefore, the final number of samples used for model construction was 979 (31 LG and 948 other conditions). The averaged preprocessed spectra for each class are shown in Figure 1D. Although the average profiles are similar, there are still visual differences between the distribution of spectra for each group, as shown in Figure 1C, where overall, the LG samples have less spread absorbance and a narrower profile.

Following preprocessing, the data were split between training (70% of samples, 22 lung cancer and 664 other conditions) and test (30% of samples, 9 lung cancer and 284 other conditions) sets. The training set was used for model construction, and the test set was used for validation. Several classification algorithms were applied to classify the data (see Supplementary Table S1), including approaches with PCA- and GA-based classifiers, PLS-DA and KNN; however, the best model for this dataset was using the PCA-QDA (principal component analysis with quadratic discriminant analysis) algorithm. The PCA-QDA model was built using eight PCs (90.4% of explained variance) and resulted in 93% accuracy for training and 91% for testing (Table 1). QDA is an excellent algorithm to handle classifications with different class sizes and, especially, with different varying structures [17,20,23]. Herein, the lung cancer class has a much smaller number of samples and a much narrower spectral distribution, indicating lower variance, while the spectra for the other condition classes are more spread, thus having a much larger variance. Hence, QDA tends to work in this scenario.

Besides good accuracy in the testing set, sensitivity was found to be 100%, indicating all lung cancer samples were correctly classified (Table 1). Specificity was found to be 91%, since some samples not identified as lung cancer were classified as such (26 out of 284 samples). In a real-world scenario, all samples identified as having lung cancer should be further investigated to avoid inaccurate diagnostics, as spectroscopy may be picking up very low-level or early disease. This is already performed since patients diagnosed with lung cancer routinely undergo CT scans or similar techniques. The F-score (the test accuracy considering the imbalanced data) and G-score (the test accuracy not accounting for the class size) at 95% indicate the different class sizes did not interfere with the model accuracy. The area under curve (AUC) for the test predictions was also calculated at 0.95, indicating excellent predictions.

**Figure 2:**
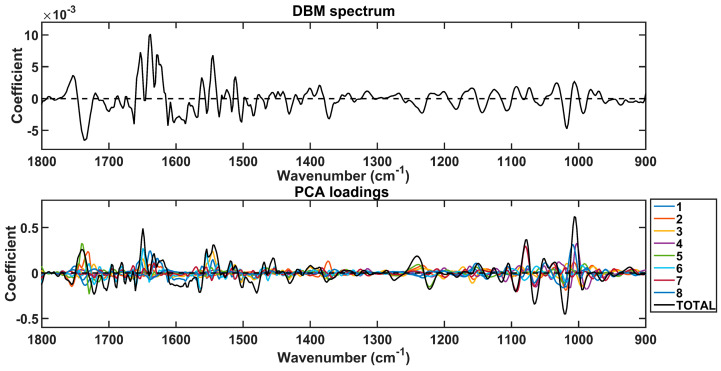
Difference-between-mean (DBM) spectrum and PCA loadings on the 8 PCs used to build the PCA-QDA model. The “TOTAL” stands for the sum of coefficients in the 8 PCs.

The spectral markers responsible for discrimination between lung cancer and other conditions were extracted based on the PCA loadings used to build the PCA-QDA model (Figure 2). These wavenumbers are listed in Table 2 along with their tentative assignments. They were selected in the regions with the largest absolute loading coefficients, matching the regions with the largest absolute coefficients in the difference-between-mean (DBM) spectrum. These regions contain the largest weights for class discrimination.

### 3.2. Prostate Cancer

**Figure 3:**
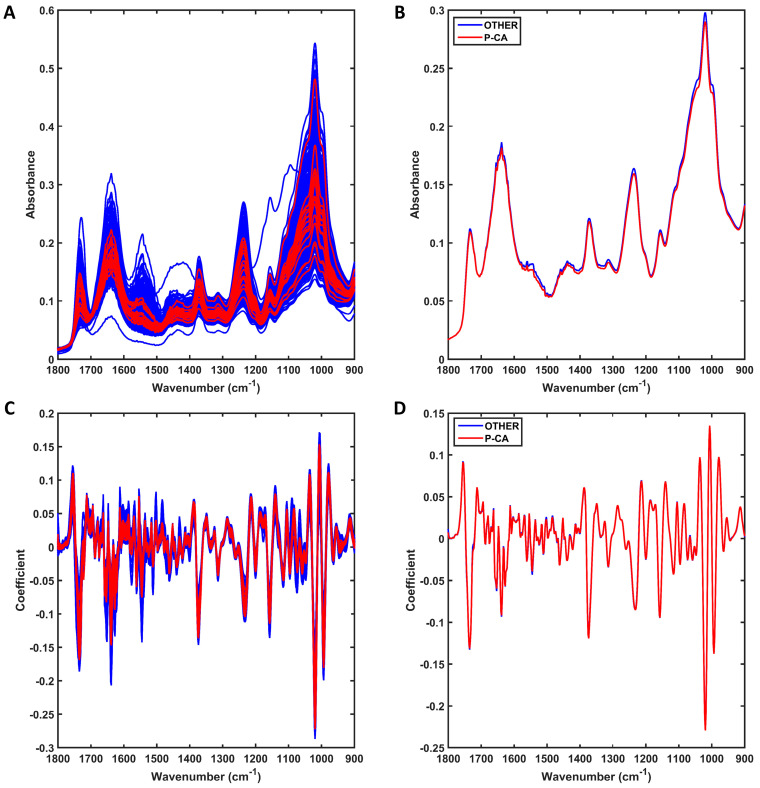
Mid-infrared (IR) spectra derived using ATR-FTIR spectroscopy. (**A**) Raw IR spectra for all samples in each group (prostate cancer [P-CA] or other conditions for male patients [OTHER]); (**B**) average raw IR spectra for each group (prostate cancer [P-CA] or other conditions for male patients [OTHER]). (**C**) Preprocessed (SG 2nd derivative followed by vector normalisation) IR spectra for all samples in each group (prostate cancer [P-CA] or other conditions for male patients [OTHER]); (**D**) average preprocessed IR spectra for each group (prostate cancer [P-CA] or other conditions for male patients [OTHER]).

The raw spectra at the fingerprint region (1800–900 cm^−1^) for the 17 prostate cancer (P-CA) samples against all remaining male patients (n = 585, OTHER) are shown in Figure 3A. Both groups of samples also share similar spectral profiles, with only small differences at approximately 1500–1650 cm^−1^ and 1000–1100 cm^−1^ as shown in the average profile per class (Figure 3B). The preprocessed spectra after outlier removal are shown in Figure 3C. For this, the spectral data were preprocessed by SG 2nd derivative (window of 9 points, 2nd-order polynomial fitting), followed by vector normalisation (Figure 3C). Outliers were removed by the Hotelling T^2^ vs. Q residuals test, where 15 outliers were identified in the other-conditions group (see Supplementary Figure S2). No P-CA outlier was observed. Therefore, the final number of samples used for model construction was 587 (17 P-CA and 570 other conditions for male patients). The averaged preprocessed spectra for each class are shown in Figure 3D. Although the average profiles are similar, there are still visual differences between the distribution of spectra for each group, as shown in Figure 3C, where overall the P-CA samples have less spread absorbance and a narrower profile.

Following preprocessing, the data were split between training (70% of samples, 12 P-CA and 399 other conditions) and test (30% of samples, 5 P-CA and 171 other conditions) sets. The training set was used for model construction and the test set was used for validation. Again, several classification algorithms were applied to classify the data (Supplementary Table S2); however, the best model for this dataset was once again using the PCA-QDA algorithm. The PCA-QDA model was built using five PCs (84.2% of explained variance) and resulted in 97% accuracy for training and 93% for testing (Table 3). QDA again showed that it works better for imbalanced data where one class has much larger variance than the second.

The prostate cancer samples were slightly better classified than the lung cancer samples. Sensitivity was found to be 100%, indicating all prostate cancer samples were correctly classified (Table 3). Specificity was found to be 92%, since some samples not identified as prostate cancer were classified as such (13 out of 171 samples). In a real-world scenario, all samples identified as having prostate cancer should also be further investigated to avoid the wrong diagnostics. This would not be a critical problem in the protocol since P-CA-diagnosed patients routinely undergo CT scans or similar techniques too. The F-score and G-score at 96% also indicate the different class sizes did not interfere with the model’s accuracy. The AUC for the test predictions was calculated at 0.96, indicating excellent predictions.

**Figure 4:**
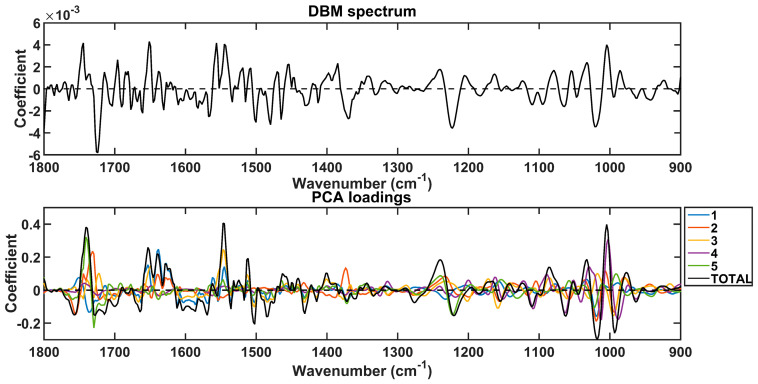
Difference-between-mean (DBM) spectrum and PCA loadings on the 5 PCs used to build the PCA-QDA model. The “TOTAL” stands for the sum of coefficients in the 5 PCs.

The spectral markers responsible for discrimination between prostate cancer and other conditions were extracted based on the PCA loadings used to build the PCA-QDA model (Figure 4). These wavenumbers are listed in Table 4 along with their tentative assignments. Again, they were selected for the regions with the largest absolute loading coefficients, matching the regions with the largest absolute coefficients in the DBM spectrum. As shown in Table 4, all the absorbances for the important wavenumbers responsible for discrimination between the classes decreased in prostate cancer.

## 4. Discussion

Early detection of cancer, especially in its asymptomatic phase, improves the prognosis [25,26]. However, the cost of a screening programme for the general population has the potential to be highly prohibitive [27]. Encouraging high levels of participation and engagement is also a challenge. Technologies that require complex manipulations are often expensive, time-consuming and intimidating to the average person; they will also typically reside in specialist centres, which adds additional expense in terms of travel for the purposes of access. There is an urgent need to exploit alternative approaches such as vibrational spectroscopy [28,29] that provide an output from the analysis of biological samples in the form of a fingerprint IR spectrum consistent with their chemical constituents and functionality. IR spectra are essentially numerical data that can then be inputted into computational algorithms [30] that can be used to diagnose a particular disease [31] or even characterise a subtype [32]. Ultimately, there will be a need to standardise the technology [33], and considerations such as substrate type will be important [4,34,35]. Human saliva would be an ideal liquid biopsy for point-of-care testing, especially in primary care settings where the patient is comfortable [36]. The advantage of ATR-FTIR spectroscopy is that it is a low-cost and robust system that is standard equipment already in pharmacy practices in many regions of the world; the major disadvantage of the overall approach is that it may take time for practitioners and regulators of new tests in clinical practice to get used to a digital read-out.

As evidenced in the Supplementary Patient Recruitment Sheet, 1000 patients participated in this trial, and except for a small number of cases, members of the public through primary care were happy to participate and provide demographic/health information; in addition, except for a very small number, the vast majority of samples were of suitable quality to allow the acquisition of IR spectra with a high signal-to-noise ratio. This suggests a protocol that is easy to implement in a typical clinical setting and has a high adherence rate. In addition to lung cancer data, other health-related data was obtained, and there were sufficient males with a high enough number of prostate cancer cases to allow us to examine the protocol as a screening test for this disease as well. The number of females and corresponding cases of breast cancer was not high enough to examine this scenario. However, our study is continuing so as to hit a target of 2000 recruits, so it is envisaged that we may be able to develop this test approach to screen for multiple disease endpoints. Such a multiplexed digital approach to screening large numbers of patients in a reagent-free, rapid and noninvasive fashion, plus the capability of readily taking repeat samplings, is hugely powerful. To determine the generalizability of our findings herein to broader populations, it will be important to expand future trials to other regions with differing socio-economic and ethnic profiles; the area in which this study was undertaken has high levels of social deprivation.

Using this spectral dataset, we found that the best model is the PCA-QDA (principal component analysis with quadratic discriminant analysis) algorithm. The PCA-QDA model is built using a small number of PCs (some 90% explained variance). QDA is an excellent algorithm for handling classifications with different class sizes and, especially, with different variance structures. All the endpoints, including accuracy, sensitivity and specificity, were exceptionally high. As expected, correlation with known outcomes is not absolutely exact and there are many reasons why this would be the case. It would be advantageous in future studies to factor in long-term follow-up of recruits to better determine if early or insidious disease is being missed by the CT scan; this might result in a better correlation with our spectrochemical approach. As our trial of this screening approach expands into other regions, it is also plausible that the detection algorithm will evolve and that PCA-QDA might be replaced. Whilst this is a large study, in respect to the general population, it is still quite modest, and numbers would need to be markedly increased to enhance the robustness of the approach. Counter-intuitively, there is also the possibility that our spectrochemical approach reported herein might be picking up minimal or early disease that is as yet undetectable or missed by conventional methods. To examine this, follow-up with these patients would be required to test for disease emergence. Of interest is the growing literature that the profile of VOCs (volatile organic compounds) in exhaled breath can indicate systemic cancer [37]. Additionally, as saliva has some 30% similarity to plasma with additional immunological factors present, it is a surprising complex biofluid. This physiological mix within saliva (hitherto largely unexplored) may allow one to use this biofluid as an alternative liquid biopsy instead of blood. The fact that breath analysis already indicates diagnostic features of cancers at distant sites such as the prostate [38] is powerful evidence in support of this.

This study presents a further expansion of our dip test [4], whereby we use a swab in saliva and analyse it using ATR-FTIR spectroscopy, and use a subsequent computational algorithm to screen for disease. Not only do we use this approach to screen for lung cancer, but we also apply the method for prostate cancer amongst male participants. The study was deliberately undertaken in a real-world clinical setting wherein the numbers of cases (i.e., disease) would be expected to be small in comparison with the overall recruitment. In an older cohort of patients sourced through primary care, our spectrochemical approach demonstrates remarkable sensitivity and specificity. As a point-of-care triage tool, this study requires verification in a multicentre trial.

## Figures and Tables

**Table 1:** Classification performance for PCA-QDA applied to classify lung cancer samples. The PCA-QDA model was built using eight PCs, accounting for 90.4% of the explained variance. The confusion matrix shows the number of samples classified in each class (other conditions or lung cancer).

	Accuracy	Sensitivity	Specificity	F-Score	G-Score
**Training**	0.93	0.73	0.93	0.82	0.82
**Testing**	0.91	1.00	0.91	0.95	0.95
**Confusion matrix (real vs. predicted class)**					
**Training**	Other conditions		LG		Total
Other conditions	620		44		664
Lung cancer	6		16		22
**Testing**	Other conditions		LG		Total
Other conditions	258		26		284
Lung cancer	0		9		9

**Table 2:** Main wavenumbers responsible for discrimination between lung cancer samples versus other conditions. The tentative assignments were made based on Movasaghi et al. [24]. ↑ indicates higher absorbance in the lung cancer class, and ↓ indicates lower absorbance in the lung cancer class.

Wavenumbers (cm^−1^)	Absorbance	Tentative Assignment
1740	↑	C=O stretching (lipids); ester C=O stretching vibration in phospholipids
1722	↑	C=O stretching vibration
1704	↑	C=O stretching vibration
1660	↓	Amide I band
1649	↓	Unordered random coils and turns of Amide I; C=O, C=N, N-H of adenine, thymine, guanine, cytosine
1633	↓	C=C stretching uracyl, C=O stretching
1620	↓	Peak of nucleic acids due to the base carbonyl stretching and ring breathing mode
1565	↓	C-C stretching (ring base)
1554	↓	C-O stretching; predominantly α-sheet of Amide II (Amide II band mainly stems from the C-N stretching and C-N-H bending vibrations weakly coupled to the C=O stretching mode)
1544	↓	Amide II band
1480	↓	Polyethylene methylene deformation modes; Amide II band
1240	↑	Asymmetric PO_2_^−^ stretching; phosphate band (phosphate stretching modes originate from the phosphodiester groups of nucleic acids); Amide III (C-N stretching mode of proteins, indicating mainly α-helix conformation)
1222	↑	Phosphate stretching bands from phosphodiester groups of cellular nucleic acids
1094	↑	Symmetric PO_2_^−^ stretching
1078	↑	Symmetric PO_2_^−^ stretching; glycogen absorption due to C-O and C-C stretching and C-O-H deformation motions
1066	↑	C-O stretching of phosphodiester and ribose
1020	↑	C-O stretching associated with glycogen
1005	↑	Ring stretching vibrations mixed strongly with CH in-plane bending

**Table 3:** Classification performance for PCA-QDA applied to classify prostate cancer (P-CA) samples. The PCA-QDA model was built using five PCs, accounting for 84.2% of the explained variance. The confusion matrix shows the number of samples classified in each class (other conditions or P-CA).

	Accuracy	Sensitivity	Specificity	F-Score	G-Score
**Training**	0.97	1.00	0.97	0.98	0.98
**Testing**	0.93	1.00	0.92	0.96	0.96
**Confusion matrix (real vs. predicted class)**					
**Training**	Other conditions		P-CA		Total
Other conditions	388		11		399
P-CA	0		12		12
**Testing**	Other conditions		P-CA		Total
Other conditions	158		13		171
P-CA	0		5		5

**Table 4:** Main wavenumbers responsible for discrimination between prostate cancer samples versus other conditions in male patients. The tentative assignments were created based on Movasaghi et al.’s work [24]. ↑ indicates higher absorbance in the prostate cancer class, and ↓ indicates lower absorbance in prostate cancer class.

Wavenumbers (cm^−1^)	Absorbance	Tentative Assignment
1740	↓	C=O stretching (lipids); ester C=O stretching vibration in phospholipids
1729	↓	C=O stretching
1715	↓	C=O stretching in thymine
1653	↓	Amide I band
1640	↓	Amide I band
1628	↓	Amide I band
1622	↓	Peak of nucleic acids due to the base carbonyl stretching and ring breathing mode
1570	↓	Amide II band
1546	↓	Amide II band
1512	↓	In-plane CH bending vibration from the phenyl rings
1503	↓	In-plane CH bending vibration from the phenyl rings
1485	↓	C_8_-H coupled with a ring vibration of guanine; CH deformation
1240	↓	Asymmetric PO_2_^−^ stretching; phosphate band (phosphate stretching modes originate from the phosphodiester groups of nucleic acids); amide III (C-N stretching mode of proteins, indicating mainly α-helix conformation)
1220	↓	PO_2_^−^ asymmetric stretching vibrations of nucleic acids when it is highly hydrogen-bonded; asymmetric hydrogen-bonded phosphate stretching mode; phosphate II (PO_2_^−^ asymmetric stretching) in *B*-form DNA
1086	↓	Symmetric phosphate stretching modes of PO_2_^−^
1062	↓	C-O stretching in deoxyribose
1033	↓	C-C stretching in skeletal *cis* conformation, CH_2_OH stretching, C-O stretching coupled with C-O bending
1018	↓	C-O stretching, C-C stretching, OCH bending in ring
1005	↓	Ring stretching vibrations mixed strongly with CH in-plane bending
993	↓	C-O stretching in ribose, C-C stretching

## Data Availability

Data contributing to this manuscript will be made available upon reasonable request to the corresponding authors.

## Supplementary Materials

The following electronic supporting information can be downloaded at: https://www.mdpi.com/article/10.3390/jpm13111533/s1, Figure S1: Outliers spectra (1800–900 cm^−1^) selected by the Hotelling T^2^ vs. Q residuals test for lung cancer (LG) vs. other conditions; Table S1: Classification performance for different algorithms applied to classify lung cancer samples; Figure S2: Outliers spectra (1800–900 cm^−1^) selected by the Hotelling T^2^ vs. Q residuals test for prostate cancer (P-CA) vs. other conditions; Table S2: Classification performance for different algorithms applied to classify prostate cancer samples. ESI Patient Recruitment Sheet.

## Abbreviations

AC, accuracy; ATR-FTIR, attenuated total reflection Fourier-transform infrared; AUC, area under curve; CT, computed tomography; DBM, difference-between-mean; FP, false positives; GA, genetic algorithm; IR, infrared; KNN, k-nearest neighbours; KS, Kennard-Stone; LDA, linear discriminant analysis; LVs, latent variables; PC, principal component; PCA, principal component analysis; PLS-DA, partial least squares discriminant analysis; QDA, quadratic discriminant analysis; RBF, radial basis function; SG, Savitzky-Golay; SENS, sensitivity; SPEC, specificity; SVN, support vector machines; TN, true negatives; TP, true positives
