# Supplementary material for: Point-of-Care Disease Screening in Primary Care Using Saliva: A Biospectroscopy Approach for Lung Cancer and Prostate Cancer
Source: J Pers Med. 2023 Oct 26;13(11):1533. doi: 10.3390/jpm13111533 (PMC10672293; doi:10.3390/jpm13111533)
Supplement: Supplementary file 1 [file jpm-13-01533-s001.zip › jpm-paper2-si-camilo.pdf]

ELECTRONIC SUPPLEMENTARY INFORMATION

Point-of-care disease screening in primary care using saliva: a biospectroscopy approach for lung cancer and prostate cancer

Francis L Martin [1,2,*], Camilo L.M. Morais[3], Andrew W Dickinson [2], Tarek Saba [2], Thomas Bongers [2], Maneesh N Singh [1,4], Danielle Bury [1,*]

[1] Biocel UK Ltd, Hull HU10 6TS, UK; mnsingh@biocel.uk (M.N.S.)
[2] Department of Cellular Pathology, Blackpool Teaching Hospitals NHS Foundation Trust, Whinney Heys Road, Blackpool FY3 8NR, UK; aw.dickinson@hotmail.com (A.W.D); dr.saba@nhs.net (T.S.); thomas.bongers@nhs.net (T.B.)
[3] Center for Education, Science and Technology of the Inhamuns Region, State University of Ceará, Tauá 63660-000, Brazil; camilomorais1@gmail.com (C.L.M.M.)
[4] Chesterfield Royal Hospital, Chesterfield Road, Calow, Chesterfield S44 5BL, UK
* Correspondence: flm13@biocel.uk (F.L.M); danielle.bury@nhs.net (D.B.)

NUMBER OF FIGURES: 2
NUMBER OF TABLES: 2

Figure S1 Outliers spectra (1800–900 cm^{-1}) selected by the Hotelling T^2 vs. Q residuals test for lung cancer (LG) *vs.* other conditions. The inset table contains the sample numbers used in this study. The label contains the outliers ID.

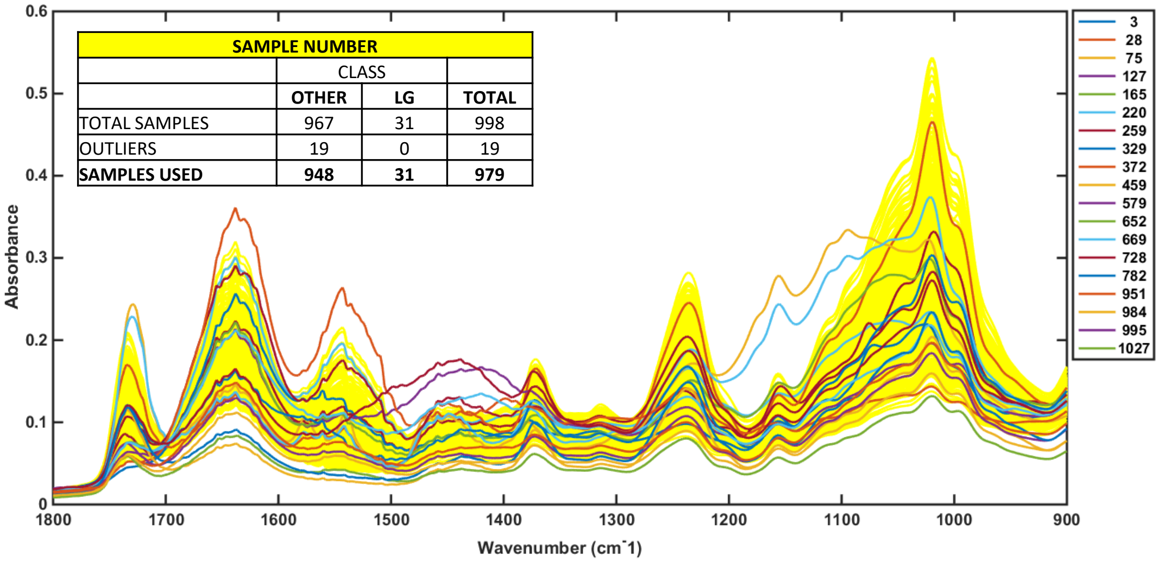

Table S1 Classification performance for different algorithms applied to classify lung cancer samples. The best model is shaded in gray. AC: accuracy, SENS: sensitivity, SPEC: specificity, F-S: F-score, G-S: G-score. PCA-LDA: principal component analysis with linear discriminant analysis, PCA-QDA: principal component analysis with quadratic discriminant analysis, PCA-SVM: principal component analysis with suppor vectors machine, GA-LDA: genetic discrimiminant analysis with linear discriminant analysis, GA-QDA: genetic algorithm with quadratic discriminant analysis, GA-SVM: genetic algorithm with support vectors machine, PLS-DA: partial least squares discriminant analysis, KNN: *k*-nearest neighbours.

		TRAINING					TESTING				
MODEL	PARAMETERS	AC	SENS	SPEC	F-S	G-S	AC	SENS	SPEC	F-S	G-S
PCA-LDA	8 PCs (90.4% EXP. VAR.)	0.61	0.68	0.60	0.64	0.64	0.29	0.67	0.29	0.40	0.44
PCA-QDA	8 PCs (90.4% EXP. VAR.)	0.93	0.73	0.93	0.82	0.82	0.91	1.00	0.91	0.95	0.95
PCA-SVM	8 PCs (90.4% EXP. VAR.) / Linear Kernel	0.95	0.00	1.00	0.00	0.00	0.97	0.00	1.00	0.00	0.00
GA-LDA	16 features	0.77	0.64	0.77	0.70	0.70	0.80	0.22	0.81	0.35	0.42
GA-QDA	16 features	0.98	0.41	1.00	0.58	0.64	0.97	0.00	1.00	0.00	0.00
GA-SVM	16 features / Linear Kernel	0.91	0.00	1.00	0.00	0.00	0.94	0.00	1.00	0.00	0.00
PLS-DA	1 LV (40% EXP. VAR.)	0.47	0.77	0.46	0.58	0.60	0.11	0.89	0.09	0.16	0.28
KNN	k=7	0.97	0.00	1.00	0.00	0.00	0.97	0.00	1.00	0.00	0.00

Figure S2 Outliers spectra (1800–900 cm^{-1}) selected by the Hotelling T^2 vs. Q residuals test for prostate cancer (P-CA) *vs.* other conditions. The inset table contains the sample numbers used in this study. The label contains the outliers ID.

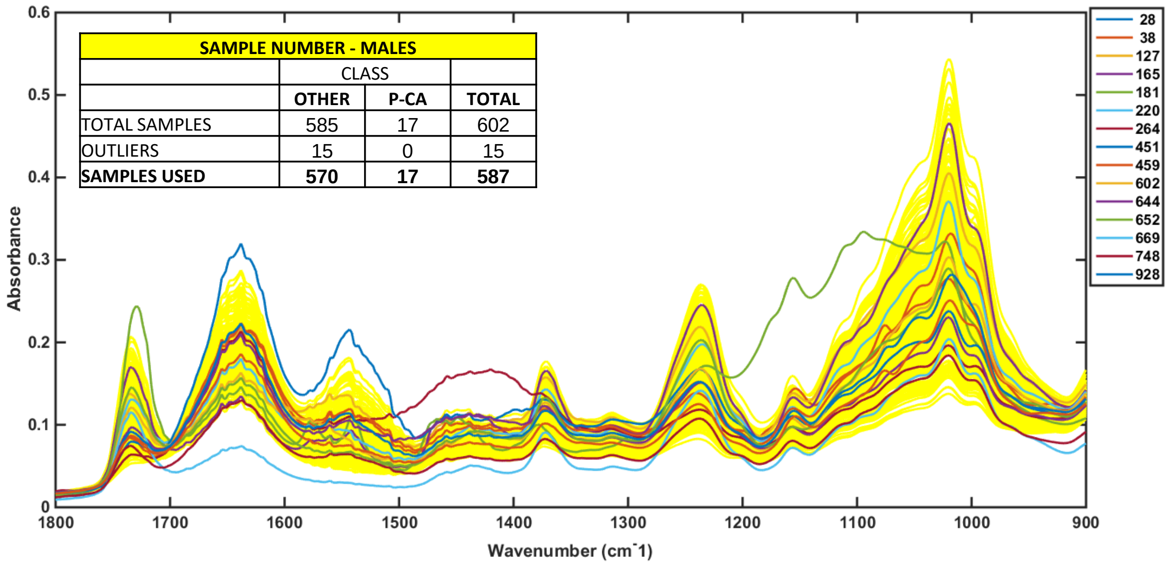

Table S2 Classification performance for different algorithms applied to classify prostate cancer samples. The best model is shaded in gray. AC: accuracy, SENS: sensitivity, SPEC: specificity, F-S: F-score, G-S: G-score. PCA-LDA: principal component analysis with linear discriminant analysis, PCA-QDA: principal component analysis with quadratic discriminant analysis, PCA-SVM: principal component analysis with suppor vectors machine, GA-LDA: genetic discrimiminant analysis with linear discriminant analysis, GA-QDA: genetic algorithm with quadratic discriminant analysis, GA-SVM: genetic algorithm with support vectors machine, PLS-DA: partial least squares discriminant analysis, KNN: *k*-nearest neighbours.

		TRAINING					TESTING				
MODEL	PARAMETERS	AC	SENS	SPEC	F-S	G-S	AC	SENS	SPEC	F-S	G-S
PCA-LDA	5 PCs (84.2% EXP. VAR.)	0.60	0.75	0.60	0.67	0.67	0.45	0.60	0.44	0.51	0.51
PCA-QDA	5 PCs (84.2% EXP. VAR.)	0.97	1.00	0.97	0.98	0.98	0.93	1.00	0.92	0.96	0.96
PCA-SVM	5 PCs (84.2% EXP. VAR.) / Linear Kernel	0.96	0.00	1.00	0.00	0.00	0.97	0.00	0.93	0.00	0.00
GA-LDA	8 features	0.71	0.58	0.72	0.64	0.65	0.81	0.00	0.83	0.00	0.00
GA-QDA	8 features	0.98	0.50	0.99	0.66	0.70	0.97	0.00	1.00	0.00	0.00
GA-SVM	8 features / Linear Kernel	0.92	0.00	1.00	0.00	0.00	0.98	0.00	1.00	0.00	0.00
PLS-DA	2 LV (51% EXP. VAR.)	0.61	0.75	0.60	0.67	0.67	0.53	0.40	0.54	0.46	0.46
KNN	k=3	0.97	0.00	1.00	0.00	0.00	0.97	0.00	1.00	0.00	0.00
